# Community assessment techniques and the implications for rarefaction and extrapolation with Hill numbers

**DOI:** 10.1002/ece3.3580

**Published:** 2017-11-21

**Authors:** Kieran D. Cox, Morgan J. Black, Natalia Filip, Matthew R. Miller, Kayla Mohns, James Mortimor, Thaise R. Freitas, Raquel Greiter Loerzer, Travis G. Gerwing, Francis Juanes, Sarah E. Dudas

**Affiliations:** ^1^ Hakai Institute Calvert Island BC Canada; ^2^ Department of Biology University of Victoria Victoria BC Canada; ^3^ Department of Biology Centre for Shellfish Research Vancouver Island University Nanaimo BC Canada; ^4^ Pacific Region Pacific Biological Station Fisheries and Oceans Canada Nanaimo BC Canada; ^5^ Centro de Estudos do Mar Universidade Federal do Paraná Pontal do Paraná Paraná Brazil; ^6^ Ecosystem Science and Management Program University of Northern British Columbia Prince George BC Canada

**Keywords:** diversity indices, ecosystem assessment, epifaunal benthic communities, Hill numbers, intertidal, methodological comparison

## Abstract

Diversity estimates play a key role in ecological assessments. Species richness and abundance are commonly used to generate complex diversity indices that are dependent on the quality of these estimates. As such, there is a long‐standing interest in the development of monitoring techniques, their ability to adequately assess species diversity, and the implications for generated indices. To determine the ability of substratum community assessment methods to capture species diversity, we evaluated four methods: photo quadrat, point intercept, random subsampling, and full quadrat assessments. Species density, abundance, richness, Shannon diversity, and Simpson diversity were then calculated for each method. We then conducted a method validation at a subset of locations to serve as an indication for how well each method captured the totality of the diversity present. Density, richness, Shannon diversity, and Simpson diversity estimates varied between methods, despite assessments occurring at the same locations, with photo quadrats detecting the lowest estimates and full quadrat assessments the highest. Abundance estimates were consistent among methods. Sample‐based rarefaction and extrapolation curves indicated that differences between Hill numbers (richness, Shannon diversity, and Simpson diversity) were significant in the majority of cases, and coverage‐based rarefaction and extrapolation curves confirmed that these dissimilarities were due to differences between the methods, not the sample completeness. Method validation highlighted the inability of the tested methods to capture the totality of the diversity present, while further supporting the notion of extrapolating abundances. Our results highlight the need for consistency across research methods, the advantages of utilizing multiple diversity indices, and potential concerns and considerations when comparing data from multiple sources.

## 1 INTRODUCTION

Effectively quantifying species diversity is a fundamental pillar in ecology. Regardless of the ecosystem, diversity estimates play a vital role in environmental monitoring (Underwood, 1994), ecosystem comparisons, anthropogenic stressor evaluation (Lovejoy, 1994), and informing conservation efforts (e.g., May, 1988). Richness and abundance estimates are two of the simplest ways to depict biodiversity and are important to consider when assessing any ecosystem (Stirling & Wilsey, 2001). They are also used to generate more complex ecological indices (Magurran, 1988), including Hill numbers, the most commonly used of which includes the diversity of all species, richness, the exponential of Shannon entropy or otherwise known as Shannon diversity, and Simpson diversity. Initially used by ecologist MacArthur (1965), and further developed and described by Hill (1973), Hill numbers have recently gone through a resurgence (see Jost, 2006, 2007; Ellison, Barker‐Plotkin, Foster, & Orwig, 2010). Hill numbers have now been extended to create an integrated approach to quantifying species diversity and abundance via sample‐ and coverage‐based rarefaction (Colwell et al., 2012; Chao & Jost, 2012; Chao et al., 2014). This modified approach allows for Hill numbers to be expressed in terms of the completeness or the proportion of individuals within an assemblage that belong to species represented in a sample (coverage) and species diversity as a function of sampling effort (Chao & Jost, 2012; Chao et al., 2014). Both of which can be extrapolated to allow ecologists to more accurately estimate species richness and further understand the differences in the diversity that exists between communities.

There is a long‐standing interest in the development of assessment techniques and improving their ability to adequately assess species diversity (Underwood, 1994; Stewart‐Oaten & Bence, 2001). Data collection is an expensive and labor‐intensive process; resource limitations place constraints on selecting the most effective approach to rigorous quantitative assessment (Oliver & Beattie, 1996; Field, Tyre, & Possingham, 2005). As a result, most community assessments still lack standardized sampling protocols and alterations to experimental design is a common occurrence (Ferrer‐Paris et al., 2013). To mitigate shortcomings, rapid, readily available, and cost‐effective techniques play an essential role in the assessment of most ecosystems (Sparrow, Sisk, Ehrlich, & Murphy, 1994; Preskitt, Vroom, & Smith, 2004) and often involve a range of sampling methods being utilized during a single assessment (Eleftheriou & Holme, 1984).

One of the original techniques used in ecological studies is quadrats, which were initially developed as a sampling unit used to detect patterns in plant communities (Greig‐Smith, 1952) and have since been used in a range of ecological studies investigating numerous habitats. Photo quadrats are also frequently used to assess various substrata by superimposing points onto photographs (Bohnsack, 1979; Preskitt et al., 2004). The flora or fauna directly underneath the points can then be identified and quantified as either abundance or percent cover. Point intercept sampling, which is very similar to photo quadrats except that it is quantified in the field, is another commonly utilized assessment method where the taxa directly underneath randomly selected points created by the intersecting grid formed by a strung quadrat are visually identified (Dethier, Graham, Cohen, & Tear, 1993; Benedetti‐Cecchi, Airoldi, Abbiati, & Cinelli, 1996). Alternatively, strung quadrats can be subdivided into smaller portions that decrease assessment time relative to evaluating the entire area (e.g., Davidson, Crook, & Barnes, 2004). Selecting a random subsample can optimize effort without compromising the validity of the results, especially if certain taxa are too abundant to be counted within a reasonable time frame (Barbour & Gerritsen, 1996).

To determine how commonly utilized substratum assessment methods capture species diversity (Hill numbers), we conducted a comparison of four methods: photo quadrats, point intercept, random subsampling, and full quadrat assessments of a smaller area (1/4 the size of the other methods). We evaluated the species density, abundance, richness, Shannon diversity, and Simpson diversity detected by each method, as well as the time each method required in situ. Furthermore, to determine the validity of each method, we conducted a method validation at a subset of the locations assessed by the previously mentioned methods. All methods were carried out in the marine intertidal, as these ecosystems have a long history of serving as model system for identifying processes that generate community patterns and structure (e.g., Paine, 1974; Lubchenco, 1978). Furthermore, soft‐sediment benthic communities have been widely used to assess and monitor natural and anthropogenic stressors (e.g., Fitch & Crowe, 2010; Gerwing, Drolet, Hamilton, & Barbeau, 2016).

We hypothesize that species density will vary according to assessment method and will be the highest when using methods that require the most effort (time). We also postulate that species abundance, once extrapolated to account for assessment area, will not vary between methods, regardless of the effort needed to conduct each assessment. The differences in species diversity and consistencies within species abundance estimates will result in similar differences within derived Shannon and Simpson diversity.

## 2 METHODS AND MATERIALS

### 2.1 Study site

**Figure 1:**
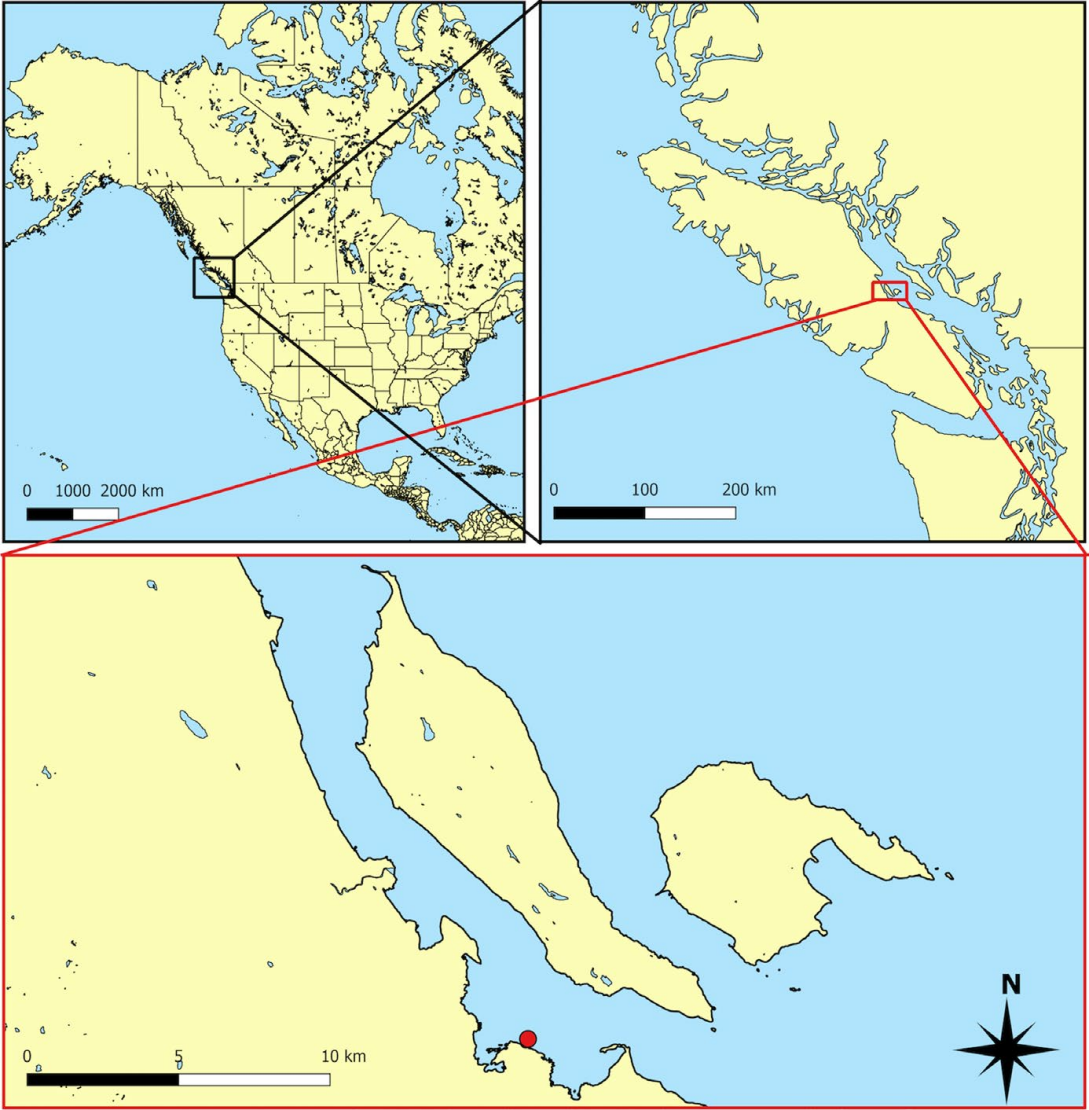
Study location in Baynes Sound, Canada (49.468417°, −124.767383°). Assessments were conducted in the intertidal ecosystems adjacent to Vancouver Island, British Columbia

This study was conducted within Baynes Sound, which is a 20 km long body of water located on the east coast of Vancouver Island, British Columbia, Canada, that consists of open shoreline, estuaries, inshore marshes, protected bays, and forests (Jamieson et al., 2001; Murray & D'Anna, 2015; Figure 1). Water circulation is primarily north to south due to flood and ebb tides and the wind‐influenced currents (Jamieson et al., 2001). Baynes Sound supports an extensive shellfish farming industry, which primarily grows Pacific oysters (*Crassostrea gigas*) and Manila clams (*Venerupis philippinarum*). The study site (49.468417°, −124.767383°) was representative of the area and consisted of a low sloping (~3%) intertidal zone comprised of soft sediments, cobble, and a relatively high abundance of bivalves.

**Figure 2:**
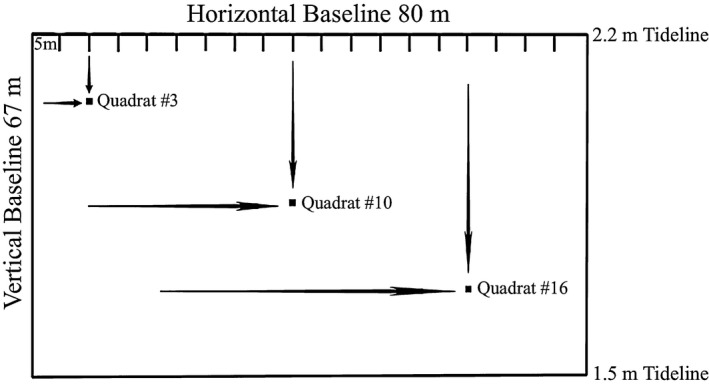
Assessment area and quadrat placements used during the methodological comparison. The 5,360 m^2^ assessment area consisted of horizontal and vertical baselines running parallel and perpendicular to the shoreline, respectively. Quadrat placement was at the intersection of perpendicular lines extended from the horizontal and vertical baselines. Examples of quadrat placement are illustrated by the placement of the 3rd, 10th, and 16th quadrats

The study site consisted of an 80 m baseline running parallel to the shore along the 2.2 m tideline, and a 67 m baseline running perpendicular to the shoreline from the 2.2 m tideline to the 1.5 m tideline, creating a 5,360 m^2^ total assessment area (Figure 2). Perpendicular lines were extended at predetermined distances along the perpendicular and parallel baselines. Assessments occurred at the intersection of these extended lines. Distances at which lines were extended along the vertical distances were chosen a priori using a random number generator, while horizontal distances increased in increments of five meters (5, 10, 15, etc.) to ensure the entire horizontal distance was covered. To decrease the risk that areas within the assessment zone were missed, an additional nine quadrats were placed at horizontal distances where selected vertical distances resulted in large gaps between adjacent quadrats. Even with these additional quadrats, none of the 26 quadrats were within five meters of each other.

Four methods were conducted at each of the 26 assessment locations, in order of least to most invasive, to decrease the risk that initial methods influenced and/or biased subsequent methods. This resulted in methods being conducted in the following order: photo quadrats, point intercept, random subsampling, and full quadrat assessments. During each assessment, all surface species (epifaunal organisms), including algae >1 mm, were identified down to the lowest reliable taxonomic unit (Norkko et al., 2002; Thrush, Hewitt, Norkko, Cummings, & Funnell, 2003; Appendix 1).

A method validation was conducted at a subset of the assessment locations (*n* = 6) during which all species within the 0.5 × 0.5 m quadrat were counted. Assessments of this nature are not logistically feasible to conduct at dozens of locations due to the time required to assess potentially thousands of individuals, as such this validation served as a baseline comparison for how well each method captured the richness and abundance present at each location.

The evaluation of the initial four methods and the method validation were considered as separate assessments. This resulted in two examinations: the results of the photo quadrats, point intercept, subsampling, and full quadrat comparison were analyzed using the data from all 26 assessment locations, and the method validation using the data collected from six of the assessment locations.

### 2.2 Method 1: Photo quadrats

Quadrats were photographed in the field using a Nikon AW120 camera held 1 m above the 0.5 × 0.5 m quadrats. Images were later cropped to the 0.5 × 0.5 m area inside the quadrat (final resolution: 2,832 ×  2,832 pixels). Fifty points were randomly generated onto each image using the software Image J (Abramoff, Magalhaes, & Ram, 2004). Organisms directly under each point were identified to the lowest possible taxonomic unit (Appendix 1).

### 2.3 Method 2: Point intercept

A 0.5 × 0.5 m quadrat with string running vertically and horizontally every 5 cm to create 100 equal squares and 81 intersecting points was used. Fifty intersections were randomly selected and organisms directly under each point were identified.

### 2.4 Method 3: Random subsample

Ten randomly selected 0.5 × 0.5 cm squares were chosen from the 100 squares formed by the strung quadrat described above. All organisms within each square were counted and identified. Abundances were multiplied by 10 to estimate total abundance.

### 2.5 Method 4: Full quadrat

A 0.25 × 0.25 m quadrat was placed in the bottom left corner of the 0.5 × 0.5 m quadrat, and all species within this area were identified. Abundance values were multiplied by four to estimate the total abundance.

### 2.6 Method validation

A 0.5 × 0.5 m quadrat assessment occurred at six of the 26 assessment locations. During this assessment, all organisms within the quadrat were counted and identified.

### 2.7 Species density, abundance, maximum richness, and assessment effort

Species density and abundance were calculated for the initial four assessment methods (*N* = 26) and the method validation (*N* = 6). As the validation method was too labor intensive to be conducted consistently, assessment effort was only calculated for the initial four methods. Species abundance consisted of the number of individuals observed during each sampling unit. The amount of time each sample took to conduct in the field was considered to be a proxy for effort. Density was calculated as the number of taxa detected in each sampling unit, while maximum richness was calculated as the total number of taxa detected by each method. Both density and richness were considered to be taxonomic density and richness, which accounts for not all organisms being identified to the species level (Gerwing, Drolet, Barbeau, Hamilton, & Allen Gerwing, 2015; Gerwing et al., 2016).

Histograms and Shapiro–Wilk tests determined that density, abundance, and sampling effort were non‐normal, despite the use of standard transformations (data not shown); as such, nonparametric tests were used. Density and assessment effort estimates were compared using Kruskal–Wallis rank sum tests and Dunn's tests to investigate differences between the methods. Total abundance was compared in the same manner as density and effort, but as the data were not comparable across all methods, photo quadrats and point intercepts, that determined abundance as individuals per assessment, were compared, and random subsampling and full quadrat assessments, that determined abundance as individuals per unit area, were compared. Additionally, random subsampling, full quadrat, and validation estimates were compared during the method validation analysis.

### 2.8 Hill numbers

Species richness, Shannon diversity, and Simpson diversity were calculated for the initial four assessment methods (*N* = 26) and the method validation (*N* = 6). Hill numbers were chosen due to their numerous advantages over other diversity indices (see Chao et al., 2014) and calculated according to Hsieh, Ma, and Chao (2016), and Chiu and Chao (2014). See Jost (2006) for a more thorough review of the benefits of Hill numbers in relation to other indices or Ellison et al. (2010) for an indication of the current consensus within the ecological community.

Richness was calculated using the following (Equation (1); Chao, 1984, 1987), where *S* denotes the number of species, *P*_*i*_ indicates the proportion of species relative to all other species detected and species are indexed by *i* = 1, 2, 3, 4. *q* denotes the sensitivity of the measure to the relative abundances and as such when q = 0, the equation considers species equally regardless of their relative abundances, which is richness (Hill, 1973; Chiu & Chao, 2014; Hsieh et al., 2016).

(1) $${}^{q}D={\left(\sum_{i=1}^{s}p_{i}^{q}\right)}^{1/(1-q)}$$

Shannon diversity, which can be considered as the number of common species in the assemblage, is frequently used in biological studies as a measure of diversity (Shannon & Weaver, 1949; Hill, 1973; Magurran, 1988; Jost, 2006; Chiu & Chao, 2014; Hsieh et al., 2016). Shannon diversity was determined for each method as denoted by Equation (2) (Hsieh, Ma, and Chao 2016). Shannon diversity is roughly equated to *q* = 1, although the equation is undefined when *q* = 1, as q approaches 1, it is the exponential of Shannon entropy (which is referred to as Shannon diversity). As *q* = 1 results in all individuals being considered equally, Shannon diversity counts species proportionately to their abundances (Chao et al., 2014).

(2) $${}^{1}D={\lim_{q\to 1}}^{q}D=\text{exp}\left(-\sum_{i=1}^{s}p_{i}^{}\text{log}pi\right)$$

Simpson diversity considers the dominant species within an assemblage while disregarding the rare species (Chiu & Chao, 2014; Hsieh et al., 2016). This occurs when *q* = 2, which is also the inverse of the Simpson concentration (Chao et al., 2014). Simpson diversity was determined for each method as denoted by Equation (3) (Good, 1953; Chao et al., 2014).

(3) $${}^{2}D=\frac{1}{\sum_{i=1}^{s}p_{i}^{2}}$$

### 2.9 Rarefaction interpolation and extrapolation

Sample‐ and coverage‐based rarefaction and extrapolation curves were generated to determine how diversity increases with increasing sampling effort and completeness. Rarefaction and extrapolation of richness, Shannon diversity, and Simpson diversity were conducted for each method according to Hsieh, Ma, and Chao (2016) and further discussed in Colwell et al. (2012), Chao and Jost (2012), and Chao et al. (2014). Sample‐based curves evaluated the number of individuals in a sample by plotting diversity estimates in relation to the number of sampling units. Coverage‐based curves were plotted against rarefied sample completeness to illustrate diversity estimates in relation to sample coverage. All extrapolation curves were plotted using a doubling in sample size, and 500 bootstrap replicates were used to estimate 95% confidence intervals. Ninety‐five percent confidence intervals, a known alternative to standard statistical testing (Magurran, 2004; Colwell, Mao, & Chang, 2004), were used to determine if differences between methods were statistically significant. Nonoverlapping 95% confidence intervals, whether rarefied or extrapolated curves are considered, indicate definite significant differences at a level <5% (Chao & Jost, 2012; Chao et al., 2014).

### 2.10 Statistical software

The analysis was conducted in R‐studio (R Core Team, 2015; R Studio Team 2015). The “dunn.test” package was used to conduct multiple nonparametric pairwise comparisons after Kruskal–Wallis rank sum tests were performed (Dunn, 1964; Dinno, 2016). Richness, Shannon diversity, Simpson diversity, and rarefaction and extrapolated curves were generated using the “iNEXT” package (Hsieh et al., 2016).

## 3 RESULTS

### 3.1 Method comparison

#### 3.1.1 Maximum richness

Sessile invertebrates and mobile invertebrates were the lowest in photo quadrats, increased during point intercept assessments and random subsampling, and were the highest during full quadrat assessments. Algal richness was consistent across methods (Table 1).

**Table 1:** Maximum taxonomic richness observed by the four assessment methods. Note that the list of the species comprising each group is available in Appendix 1

	Photo quads	Point intercept	Subsampling	Full quads
Algae	3	3	3	3
Sessile invertebrates	2	2	4	4
Mobile invertebrates	3	7	13	15
Maximum observed diversity	8	13	20	22

#### 3.1.2 Assessment effort

**Figure 3:**
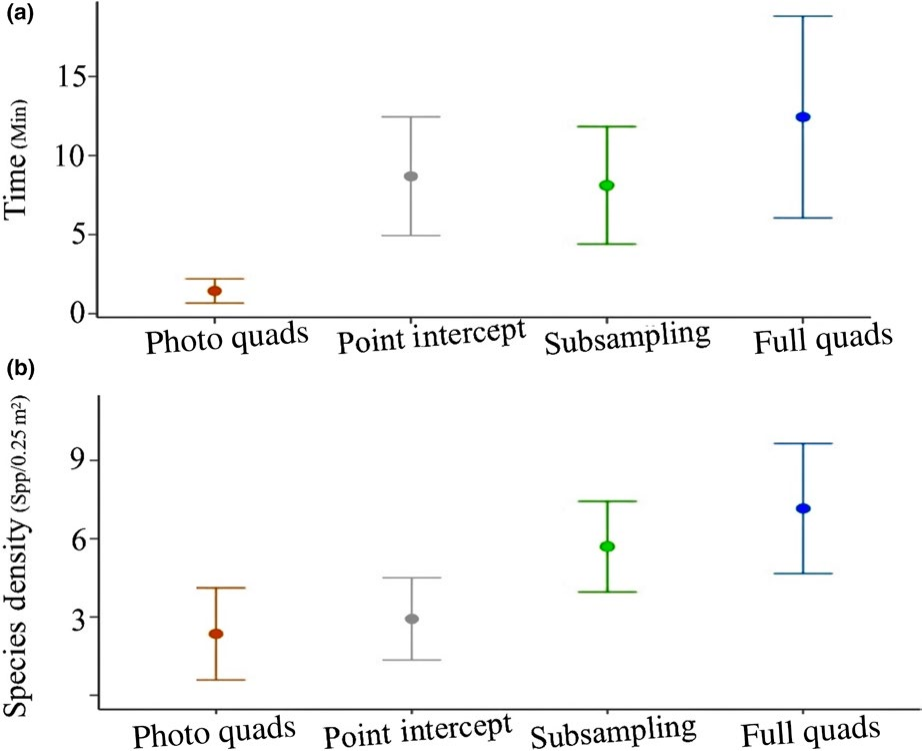
Results of Kruskal–Wallis rank sum tests determining whether the time needed to conduct the assessment (effort) and the number of species observed varied among the assessment methods. (a) Assessment effort (N = 26). Kruskal–Wallis rank sum test chi‐squared= 63.97, df = 4, p < .001. (b) Species density (N = 26). Kruskal–Wallis chi‐squared = 68.09, df = 4, p < .001

The amount of field time required to complete each assessment was the lowest in photo quadrats and increased during point intercept, random subsampling, and full quadrat assessments (Figure 3; Kruskal–Wallis $\chi_{4}^{2}$ **= 63.97, *p* < .01). Photo quadrats took significantly less field time than any other method. The time needed to conduct point intercept assessments did not differ significantly from that of random subsampling or full quadrat assessments. Random subsampling took significantly less time than full quadrat assessments (Figure 3; Table 2)

**Table 2:** Results of separate Dunn’s test analyses conducting nonparametric multiple pairwise comparisons to determine if the time (effort) needed to conduct the assessment or the number of taxa (species density) detected during each assessment varied between methods. The Dunn's tests were conducted post hoc following a Kruskal–Wallis rank sum test

	Photo quads	Point intercept	Subsampling
Effort			
Point intercept	0.000**		
Subsampling	0.000**	0.379	
Full quads	0.000**	0.071	0.033*
Species Density			
Point intercept	0.253		
Subsampling	0.000**	0.000**	
Full quads	0.000**	0.000**	0.085

Significant *p* values <.05 are indicated via *, *p* values <.01 are indicated via **.

#### 3.1.3 Species density

The species density detected by each method increased in the order in which the assessments were carried out. Density estimates were the lowest in photo quadrats, increased during point intercept assessments, further increased in random subsampling, and were the highest in full quadrat assessments (Figure 3; Kruskal–Wallis $\chi_{4}^{2}$ = 68.09, *p* < .01). The density detected by subsampling and full quadrat assessments was significantly higher than the richness detected by photo quadrats or point intercepts. However, increases in density were not significant between photo quadrats and point intercept assessments, and random subsampling and full quadrat assessments (Figure 3; Table 2).

#### 3.1.4 Species abundances

**Figure 4:**
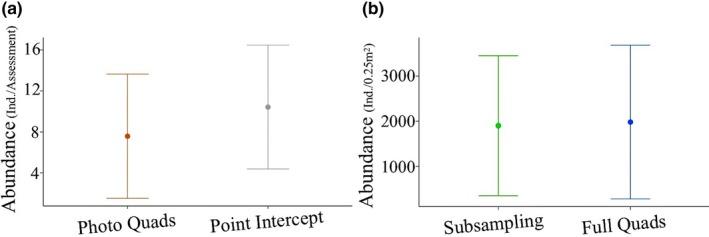
Results of Kruskal–Wallis rank sum tests determining whether species abundances varied between assessment methods. (a) Photo quadrats and point intercepts comparison (*N* = 26). Kruskal–Wallis rank sum test chi‐squared = 2.209, *df* = 1, *p* > .1. (b) Random subsampling and full quadrat assessments comparison (*N* = 26). Kruskal–Wallis chi‐squared = 0.0121, *df* = 1, *p* > .1

Abundance estimates varied marginally during either photo quadrat and point intercept comparisons or random subsampling and full quadrat comparisons. There was no statistical difference between species abundance observed by photo quadrats or point intercepts (Figure 4; $\chi_{1}^{2}$ = 4.15 *p* > .1). Additionally, there was no significant difference between abundances detected by random subsampling and full quadrat assessments, once the initial values were extrapolated to determine the number of individuals likely present within the 0.5 × 0.5 m area ($\chi_{1}^{2}$ = 0.01, *p* > .1).

#### 3.1.5 Hill numbers

**Figure 5:**
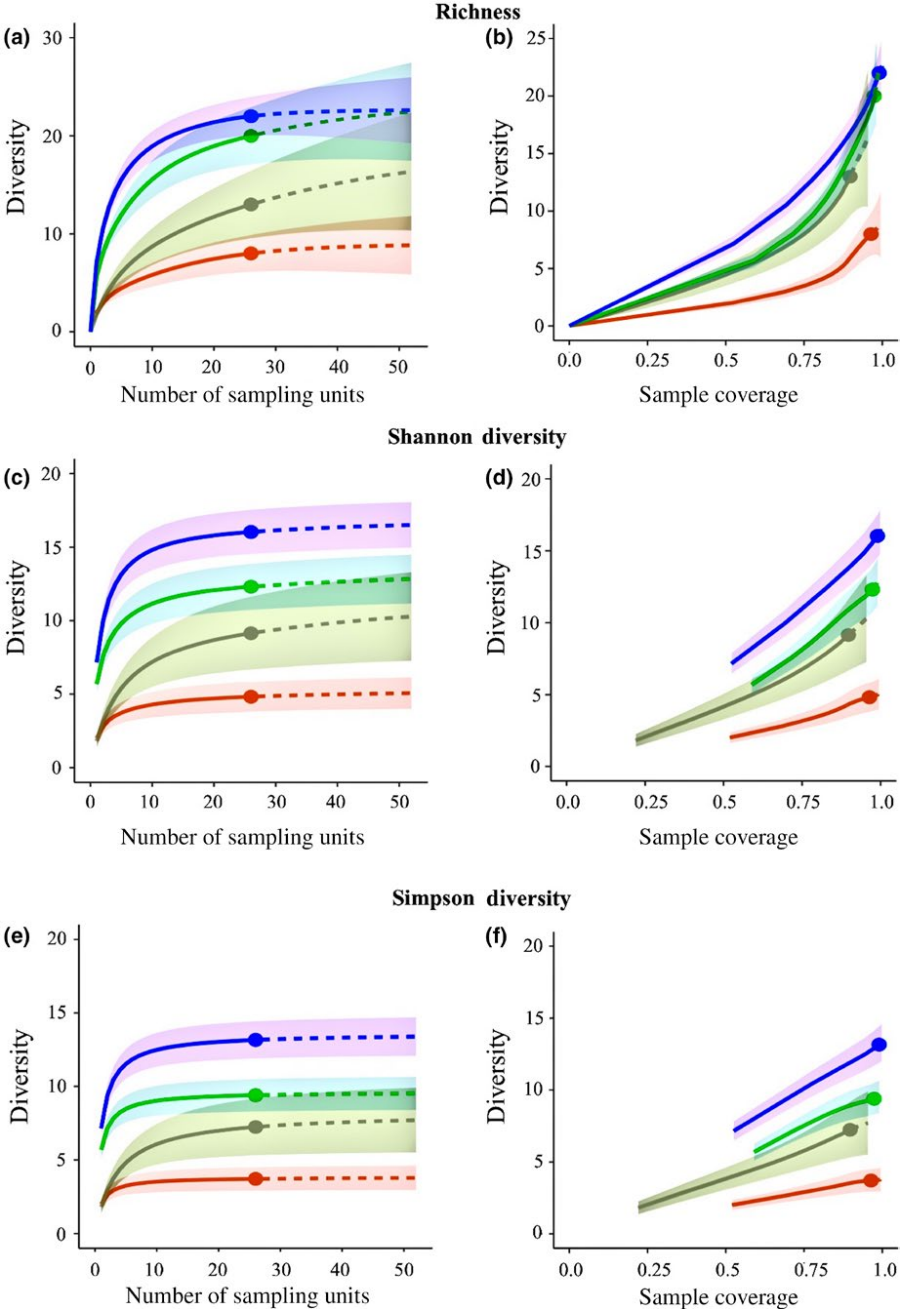
Four method comparison using sample‐ and coverage‐based rarefaction and extrapolation of Hill numbers. Orange = photo quadrats, gray = point intercept, green = subsampling, blue = full quadrat. (a) Richness (*q* = 0) sample‐based rarefaction and extrapolation, (b) richness (*q* = 0) coverage‐based rarefaction and extrapolation, (c) Shannon diversity (*q* = 1) sample‐based rarefaction and extrapolation, (d) Shannon diversity (*q* = 1) coverage‐based rarefaction and extrapolation, (e) Simpson diversity (*q* = 2) sample‐based rarefaction and extrapolation, (f) Simpson diversity (*q* = 2) coverage‐based rarefaction and extrapolation. All extrapolation curves were plotted to a doubling in sample size, and 500 bootstrap replicates were used to estimate 95% confidence intervals

Detection rates, as well as the total number of species detected by the sample‐ and coverage‐based rarefaction and extrapolation curves, increased in the order in which the assessments methods were carried out. Both curves indicated that richness estimates were the lowest in photo quadrats, increased during point intercept assessments, further increased in random subsampling, and were the highest in full quadrat assessments (Figure 5). During the sample‐based rarefaction curves, richness estimates detected by subsampling and full quadrat assessments were significantly higher than the richness detected by photo quadrats or point intercepts; however, during extrapolation, the 95% confidence intervals converged so that point intercepts, subsampling, and full quadrat assessments did not differ significantly (Figure 5). A similar trend was observed between photo quadrats and point intercepts assessments, as they only slightly overlapped during rarefaction, implying a significant difference in diversity at most sampling efforts, but during extrapolation, the 95% confident intervals converged. Additionally, nonoverlapping confidence intervals indicated that at numerous sampling efforts, especially with low amounts of sampling effort, significant differences between the numbers of species detected by the various methods existed. Coverage‐based rarefaction and extrapolation curves indicated that sample coverage (completeness) was above 90% during all methods, implying that correcting for sample completeness is likely not warranted as the lowest coverage, known as the base coverage, did not differ drastically from the highest coverage value.

Much like richness, Shannon and Simpson diversities detected by the sample‐ and coverage‐based rarefaction and extrapolation curves increased according to the order in which the assessment methods were carried out. Again, both curves indicated that Shannon diversity and Simpson diversity were the lowest in photo quadrats, increased during point intercept assessments, further increased in random subsampling, and were the highest in full quadrat assessments (Figure 5). However, unlike richness, Shannon diversity and Simpson diversity estimates detected by sample‐based rarefaction and extrapolation curves were statistically higher during full quadrat assessments than any of the other methods, and photo quadrats were statistically lower than the three other methods. Much like richness estimates, coverage‐based rarefaction and extrapolation indicated that sample completeness was relatively consistent across methods as coverage values were all over 95%, and in most cases, confidence intervals did not overlap.

The vast majority of the sample‐based rarefaction and extrapolation curves assessing richness, and all of the curves addressing Shannon and Simpson diversity, plateaued during the 26 quadrat assessments (Figure 5). Plateauing richness curves suggests that each method reached its detection limits and the majority of species that could be detected using each method were indeed identified, despite undetected species still being present within the ecosystem. Plateauing Shannon and Simpson diversity indicated that each assessment method reached the maximum value for these metrics given the diversity and abundance present within the ecosystem and each method's detection capabilities.

### 3.2 Method validation

#### 3.2.1 Richness and abundances estimates

**Figure 6:**
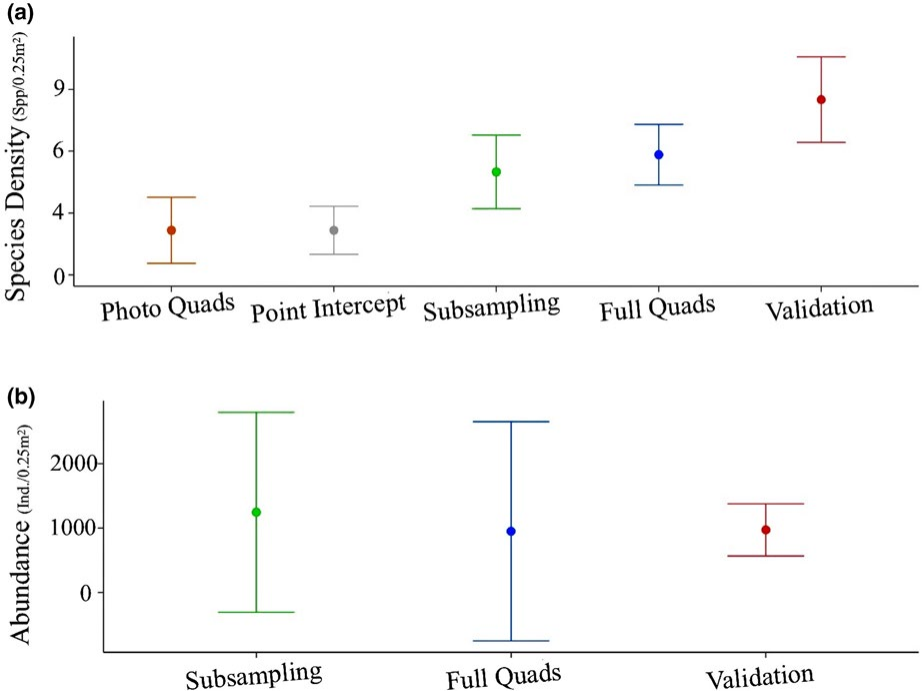
Results of Kruskal–Wallis rank sum tests determining whether species abundances and richness determined during the method validation varied between assessment methods. (a) Species density. Kruskal–Wallis chi‐squared = 21.09, df = 4, *p* < .001 (b) Species abundance. Kruskal–Wallis chi‐squared = 1.06, *df* = 2, *p* > .05

Abundances observed during the method validation did not differ from those determined by random subsampling or full quadrat assessments (Figure 6; Table 3). The species richness detected by the method validation was significantly higher than the richness detected by photo quadrats, point intercept, or random subsampling. Although validation assessments detected higher richness than the full quadrat assessments, the increase was not significant (Table 3).

**Table 3:** Results of separate Dunn test analyses conducting nonparametric multiple pairwise comparisons to determine if abundances and species density varied between methods during the method validation (*N* = 6). The Dunn's tests were conducted post hoc following a Kruskal–Wallis rank sum test

	Photo quads	Point intercept	Subsampling	Full quads
Abundance				
Full quads			0.1790	
Validation			0.4784	0.1935
Species Density				
Point intercept	0.5000			
Subsampling	0.0268*	0.0268*		
Full quads	0.0058**	0.0058**	0.2762	
Validation	0.0001**	0.0001**	0.0374*	0.1174

Significant *p* values <.05 are indicated via *, *p* values <.01 are indicated via **.

#### 3.2.2 Hill numbers

**Figure 7:**
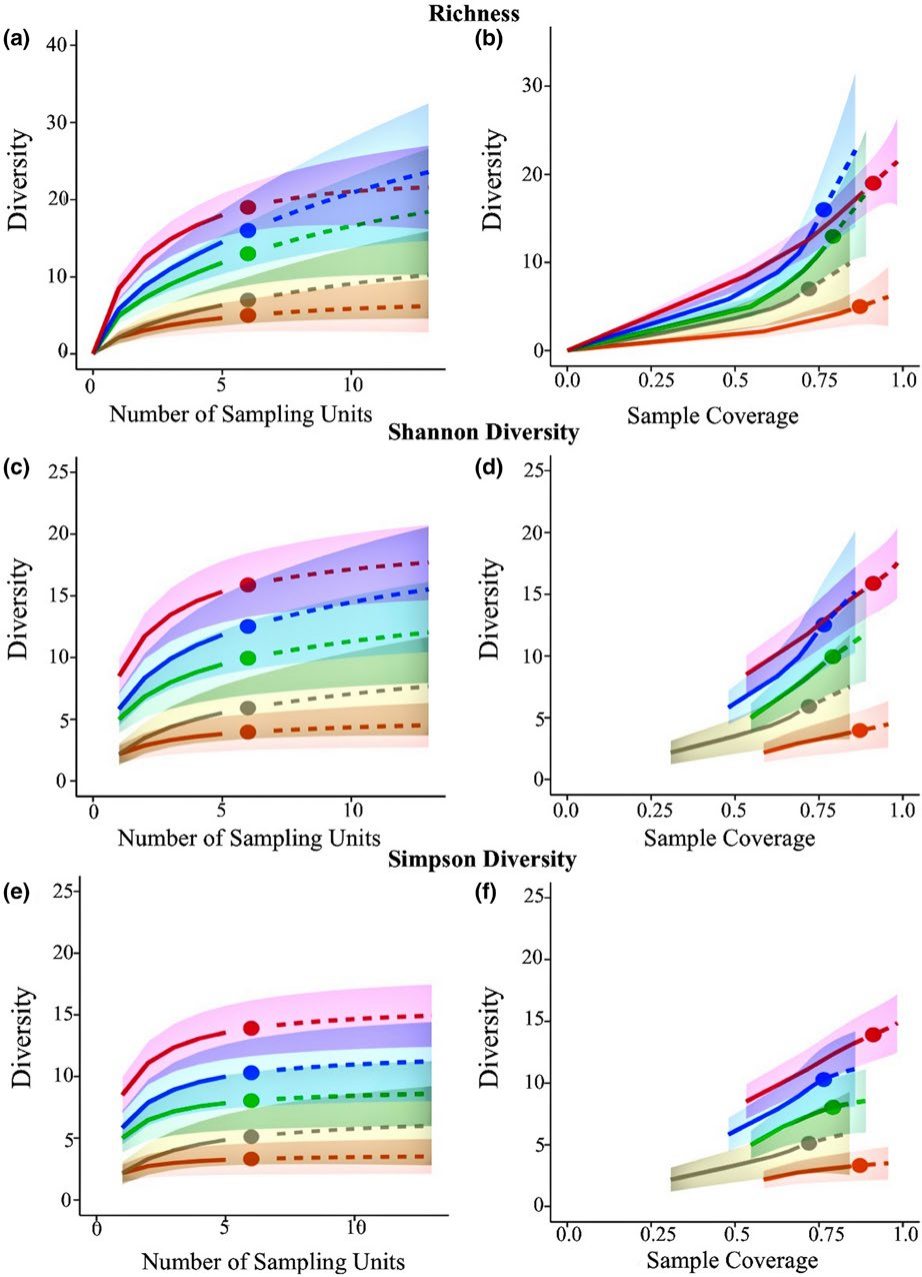
Method validation using sample‐ and coverage‐based rarefaction and extrapolation of Hill numbers. Orange = photo quadrats, gray = point intercept, green = subsampling, blue = full quadrat, red = validation. (a) Richness (*q* = 0) sample‐based rarefaction and extrapolation, (b) richness (*q* = 0) coverage‐based rarefaction and extrapolation, (c) Shannon diversity (*q* = 1) sample‐based rarefaction and extrapolation, (d) Shannon diversity (*q* = 1) coverage‐based rarefaction and extrapolation, (e) Simpson diversity (*q* = 2) sample‐based rarefaction and extrapolation, (f) Simpson diversity (*q* = 2) coverage‐based rarefaction and extrapolation. All extrapolation curves were plotted to a doubling in sample size, and 500 bootstrap replicates were used to estimate 95% confidence intervals

The sample‐ and coverage‐based rarefaction and extrapolation curves of the validation method had the highest number of observed species as well as the most predominant detection rate compared to the other methods (Figure 7). However, increases in richness detected by sample‐based rarefaction and extrapolation were only significant relative to photo quadrat and point intercept assessments as confidence intervals for random subsampling, full quadrat assessments, and the validation method converged during rarefaction and more so during extrapolation. Coverage‐based curves indicated that sampling method coverages were above 80% in all cases, with the validation method being the only method with 100% coverage. Given the consistency of coverage values across methods and the notion that even if all methods were scaled to the base coverage value, the order of species detected would not change, the methods' diversity estimates were not corrected based on their coverage.

Shannon and Simpson diversity estimates determined by the sample‐ and coverage‐based rarefaction and extrapolation curves were higher in the validation than any other method (Figure 7). However, these increases in Shannon diversity were only significant when comparing the validation method to photo quadrats and point intercept assessments, and increases in Simpson diversity were only significant when comparing the validation method to photo quadrats, point intercept, and subsampling assessments. Much like richness, coverage‐based curves detected coverage values that ranged from 80% to 100%, with the validation method being the only method to reach 100% coverage. Again, as coverage values did not differ drastically and reducing all Shannon and Simpson diversity estimates to the base coverage would not alter the hierarchy of the assessments methods, comparing methods at their current coverage values was deemed appropriate.

Of the sample‐based rarefaction and extrapolation curves evaluating increases in richness with additional sampling effort, only photo quadrats and the validation method plateaued within the extrapolation curve. However, all methods plateaued if extrapolation was increased from a doubling to a quadrupling (i.e., 24 sampling units), while the relative order of the methods remained unchanged (data not shown) indicating that the detection capabilities of each method was reached or was within reach given the sampling effort. Similarly, to the method comparison, the majority of the sample‐based rarefaction and extrapolation curves evaluating Shannon and Simpson diversities plateaued within the extrapolation curve, and those that did not plateau, did, if the extrapolation curve was extended. Again, this implies that the maximum Shannon and Simpson diversity values possible were reached given the detection capabilities of each method, and the diversity and abundance within the ecosystem were reached.

## 4 DISCUSSION

To determine the relative effectiveness of commonly used substratum assessment methodologies, as well as their implications for diversity indices, we conducted a comparison of four assessment methods and derived Hill numbers. A method validation was also conducted to determine how well each method captured the diversity present at each location.

### 4.1 Method comparison

Species density, maximum richness, and assessment effort were the highest in the full quadrat assessments, which was the most invasive, labor‐intensive method, and the lowest during photo quadrats, which was the least invasive of the methods. However, the increase in the density of species detected by full quadrat assessments relative to random subsampling was not significant, but the additional time needed to conduct full quadrat assessments was significant. These findings suggest that the average number of species detected and the effort needed to conduct the assessments increased in a similar fashion until detection rates plateaued despite further increases in effort. Stabilizing detection rates are likely a function of the detection capabilities of each method. Therefore, if field assessments are required, random subsampling would be preferential to full quadrat use as it detects similar densities in less time. Additionally, density estimates detected by photo quadrats and point intercepts did not differ significantly, but the effort needed to conduct point intercept was significantly higher; thus, if time in the field is a limiting factor, photographing substrata for later analysis is likely an appropriate course of action. However, it is worth noting that the time required to process images was not included in the comparison and any costs that may be incurred during image processing must be considered before deeming photo quadrats a suitable alternative to point intercept assessments. Variation between methods highlights an observation made decades ago by May (1988) and later elaborated by Gotelli and Colwell (2001) that although diversity is a natural measurement, it can be quite difficult to quantify properly. Subsequently, if the assessment methods result in errors in species detection it is possible to underestimate the occurrence of common species (Gu & Swihart, 2004). Unfortunately, given the low species detection rates within photo quadrats or point intercept assessments, this may be the case in studies utilizing these methodologies to sample anything but flora. These results are especially concerning for studies comparing multiple data sets, data collected under varying conditions or assessments that use multiple methods. Any differences in communities assessed under these circumstances may be attributed to differences in diversity but could also be the result of variations in the methods or effort.

Due to the differences between the methodologies, abundance estimates were separated into a comparison of photo quadrats and point intercepts, and random subsampling and full quadrat assessments. Both comparisons indicated that extrapolated abundances result in comparable estimates. Additionally, point intercepts took significantly longer than photo quadrats, and full quadrat assessments took significantly longer than random subsampling without significant increases in abundances being detected in either case, further supporting the notion that increases in effort are not necessarily associated with increases in detection rates. Based on this observation, photo quadrats are preferential to point intercept assessments, if photo‐processing time is not a concern, and random subsampling is preferential to full quadrat assessments. Moreover, extrapolating abundances may decrease assessment effort without altering the quality of the estimate. These results support past studies in which abundance estimates were extrapolated based on subsample estimates (Kunin, 1998). As species abundance is commonly used to generate more complex indices and inform conservation efforts (Caughley & Gunn, 1996), these findings help to validate index generation by showing consistency within abundance estimates across different methods. This supports the use of abundance estimates and derived indices when making ecologically relevant decisions.

Although not a formal cost‐benefit analysis, the finding that species detection rates plateaued despite increases in effort and that extrapolating abundances is an appropriate course of action suggests that concerns surrounding the validity of subsampling techniques although understandable are likely not justified (Barbour & Gerritsen, 1996; Lorenz, Kirchner, & Hering, 2004). Furthermore, this comparison provides the framework for conducting a statistically credible and cost‐effective ecological assessment.

Sample‐based rarefaction and extrapolation curves of richness, Shannon, and Simpson diversities indicated that each method had a significantly higher detection rate than the previous methods, the majority of these increases being statistically significant. The curves of each method matched the previously determined maximum species richness, indicating that these curves can provide reliable estimates of total species richness, a fact that despite growing evidence (Thompson & Withers, 2003) is still under debate (He & Hubbell, 2011). As the majority of the curves plateaued, especially when extrapolation was considered, it can be assumed the differences between curves are a function of the differences between the sampling processes and their detection rates, not a lack of sampling effort (Chao & Jost, 2012). It is worth noting that this finding is not well addressed when discussing rarefaction, extrapolation, or any comparable species accumulation curves. The traditional viewpoint is that if curves plateau then the majority of the species within the system have been detected. (See Schloss & Handelsman, 2004; Olszewski, 2004; Tringe et al., 2005; Colwell, 2009). However, as these results highlight, the majority of curves plateaued, despite species still being present within the ecosystem. Thus under most circumstances, plateauing, regardless of extrapolation, does not indicate that the majority of species have been detected, but instead indicates the detection capabilities of the method have been reached.

Coverage‐based rarefaction and extrapolation curves of richness, Shannon, and Simpson diversities indicated that the majority of assessment methods have similar relative abundances of observed species (Chao et al., 2014), with all four methods reporting over 90% coverage (completeness). Under these circumstances, accounting for the difference in coverages by reducing all methods to the base coverage was not warranted. However, this analysis does highlight the need for coverage‐based rarefaction and extrapolation, as initially highlighted by Alroy (2010) and Jost (2010), and further developed by Chao and Jost (2012). Comparing coverage‐based curves allows for the degree to which diverse communities differ to be attributed to differences between those communities and not sampling effort. Although in this instance a standard coverage was not necessary, coverage‐based curves further solidified the notion that observed differences between communities are due to differences between the assessment methods, not the assessment effort. Future studies addressing the differences between assessment methodologies, especially cost‐benefit analyses, could benefit from including a coverage‐based stopping principle to allow for sampling to be conducted until a predetermined level of coverage. Methodologies compared at a level of equal completeness, not sample size, would allow for further insight into the differences between communities rather than samples (Chao & Jost, 2012; Rasmussen & Starr, 1979).

### 4.2 Method validation

During the method validation, no significant differences in abundances estimates were detected between random subsampling, full quadrats, and validation assessments. The lack of variation between extrapolated abundances supports the notion that using a method that decreasing assessment effort by extrapolating abundances is likely an acceptable practice (Kunin, 1998). However, species density estimates were much higher in validation assessments, and with the exception of full quadrat assessments, the increased density was significant in all cases. The lack of difference between the full quadrat and validation assessments may be a function of the small sample sizes or may indicate that full 1/4th quadrat assessments adequately captured the species present despite their reduced size. The differences between the four methods and the validation assessment highlight that in all cases, species density estimates were lower than what is possible if assessment effort is not a concern. This result is concerning, as numerous studies have used these or similar methodologies and may have failed to capture the totality of the richness present.

Sample‐ and coverage‐based rarefaction and extrapolation curves showed a similar trend to the previous four method comparison, as richness, Shannon diversity, and Simpson diversity were all higher in the validation method than any of the other assessment methods. Although the statistical significance of these increases varied somewhat according to the method, they still indicated that diversity estimates, detection rates, and sample completeness were lower than what is possible if assessment effort is not a concern. Again, the majority of the sample‐based curves plateaued, which may indicate that the majority of species within the ecosystem have been detected (see Schloss & Handelsman, 2004; Olszewski, 2004; Tringe et al., 2005; Colwell, 2009); however, as the initial method comparison illustrated, this is not the case and each method has simply reached its detection capabilities.

## 5 CONCLUSION

To determine how well commonly used substratum assessment methodologies capture species diversity, we conducted a methodological comparison using four assessment methods and derived Hill numbers. A method validation was also conducted to determine how well each method captured the total diversity present at each location. Results indicated that species density, richness, Shannon diversity, and Simpson diversity vary significantly between methods, while abundance estimates do not. Under these conditions, random subsampling was preferential to photo quadrats, point intercepts, or full quadrat assessments, in terms of species detected and effort required. Coverage‐based curves confirmed that differences between assessment methods were not due to varying levels of completeness between methods, but instead fundamental differences between the detection capabilities of each method.

Ecosystem assessments should consider methodologies that seek to minimize sampling effort through subsampling or extrapolating and whenever possible indices should be used in conjunction with each other. These findings provide the framework necessary to effectively quantify species across a range of ecosystems, further the development of readily available, cost‐effective techniques, and the efficient use of ecological indices to portray ecological trends, all of which are fundamental to the application and preservation of ecology.

## CONFLICT OF INTEREST

None declared.

## AUTHOR CONTRIBUTION

This manuscript was a collaborative effort between the listed co‐authors, which involved the listed authors being involved in project design, data collection, literature review, and multiple manuscript revisions. As the lead author, Kieran Cox conducted the analysis, under the supervision of Dr. Sarah Dudas, Dr. Travis Gerwing, and Dr. Francis Juanes. All listed authors were vital to the completion of this manuscript.

## APPENDIX 1

### Species diversity (Mean ± standard error) detected by the five assessment methodologies used during this study

**Table:**

	Photo quads	Point intercept	Subsampling	Full quads	Validation
Sessile invertebrates					
*B. glandula*	0	6.12 ± 0.9	142.46 ± 23.8	344.92 ± 66.2	706.50 ± 112.2
*C. dalli*	0	0	0.19 ± 0.1	0.88 ± 0.6	1.83 ± 1.2
*Mytilus* spp.	0	0	0.35 ± 0	1.15 ± 0	0.83 ± 0.7
*Balanus* spp.	4.42 ± 0.7	0	0	0	0.33 ± 0.3
*C. gigas*	1.35 ± 0.3	1.50 ± 0.4	0.19 ± 0.1	0.69 ± 0.3	1.50 ± 1.0
*A. Artemisia*	0	0	0	0.04 ± 0	0
Mobile invertebrates					
*M. columbiana*	0	0	0.60 ± 0.2	2.77 ± 0.9	4.50 ± 2.0
*T. persona*	0	0.50 ± 0.2	7.85 ± 2	27.12 ± 6	47.33 ± 19.0
*P. torva*	0	0	0	0	0.17 ± 0.2
*L. sitkana*	0	0.04 ± 0	2.04 ± 0.6	3.46 ± 1.0	13.33 ± 7.2
*H. oregonensis*	0	0.19 ± 0.1	2.31 ± 0.5	6.54 ± 2.0	7.50 ± 2.2
P. spp	0	0	0	1.00 ± 0.5	0.33 ± 0.3
*Hemigrapsus* spp	0	0.46 ± 0.2	12.50 ± 4.2	29.69 ± 5.1	57.83 ± 36.4
*B. attramentaria*	0	0.58 ± 0.3	0.88 ± 0.4	12.73 ± 11.4	3.17 ± 1.7
*Nucella* spp.	0.08 ± 0.1	0	0.12 ± 0.1	0.54 ± 0.2	0.83 ± 0.7
*L. scutulata*	0	0	0.69 ± 0.4	2.54 ± 1.4	7.50 ± 6.6
*Littorina* spp	0.04 ± 0	0.04 ± 0	0.12 ± 0.1	0	0.50 ± 0.5
*H*. *nudus*	0	0.04 ± 0	0.04 ± 0	0.65 ± 0.3	0
*P. caurinus*	0	0	0	0.04 ± 0	0
*I. wosnesenskii*	0	0	0.35 ± 0.3	0.58 ± 0.5	0
*V. philippinarum*	0	0	0	0.08 ± 0.1	0
*P. peregrina*	0	0	0.04 ± 0	0.08 ± 0.1	0
*U. pugettensis*	0	0	0.04 ± 0	0	0
*T. scutum*	0.12 ± 0.1	0	0	0	0
Algae					
*Hildenbrandia* spp.	0	0	0	40.38 ± 19.2	64.67 ± 64.7
*Gracilaria* spp	0.04 ± 0	0.58 ± 0.2	6.54 ± 4.9	18.38 ± 10.7	52.00 ± 33.3
*Ulva* spp.	0.23 ± 0.2	0.08 ± 0.1	0	0	0.50 ± 0.5
*M. jardinii*	0	0.12 ± 0.1	9.92 ± 5.7	0	0
*Ceramium* spp	0.65 ± 0.2	0.19 ± 0.2	3.00 ± 2.1	1.27 ± 1.1	0
*E. muricata*	0.65 ± 0.2	0.00 ± 0	0.00 ± 0	0	0
